# Differentiation between Phyllodes Tumors and Fibroadenomas through Breast Ultrasound: Deep-Learning Model Outperforms Ultrasound Physicians

**DOI:** 10.3390/s23115099

**Published:** 2023-05-26

**Authors:** Zhaoting Shi, Yebo Ma, Xiaowen Ma, Anqi Jin, Jin Zhou, Na Li, Danli Sheng, Cai Chang, Jiangang Chen, Jiawei Li

**Affiliations:** 1 Department of Oncology, Shanghai Medical College, Fudan University, No. 270, Dong’an Road, Xuhui District, Shanghai 200032, China; 2 Department of Medical Ultrasound, Fudan University Shanghai Cancer Center, No. 270, Dong’an Road, Xuhui District, Shanghai 200032, China; 3 Shanghai Key Laboratory of Multidimensional Information Processing, School of Communication and Electronic Engineering, East China Normal University, No. 500, Dongchuan Road, Shanghai 200241, China; 4 Department of Radiology, Fudan University Shanghai Cancer Center, No. 270, Dong’an Road, Xuhui District, Shanghai 200032, China; 5 Engineering Research Center of Traditional Chinese Medicine Intelligent Rehabilitation, Ministry of Education, No. 1200, Cailun Road, Pudong District, Shanghai 201203, China

**Keywords:** phyllodes tumor, fibroadenoma, deep learning, ultrasound, breast

## Abstract

The preoperative differentiation of breast phyllodes tumors (PTs) from fibroadenomas (FAs) plays a critical role in identifying an appropriate surgical treatment. Although several imaging modalities are available, reliable differentiation between PT and FA remains a great challenge for radiologists in clinical work. Artificial intelligence (AI)-assisted diagnosis has shown promise in distinguishing PT from FA. However, a very small sample size was adopted in previous studies. In this work, we retrospectively enrolled 656 breast tumors (372 FAs and 284 PTs) with 1945 ultrasound images in total. Two experienced ultrasound physicians independently evaluated the ultrasound images. Meanwhile, three deep-learning models (i.e., ResNet, VGG, and GoogLeNet) were applied to classify FAs and PTs. The robustness of the models was evaluated by fivefold cross validation. The performance of each model was assessed by using the receiver operating characteristic (ROC) curve. The area under the curve (AUC), accuracy, sensitivity, specificity, positive predictive value (PPV), and negative predictive value (NPV) were also calculated. Among the three models, the ResNet model yielded the highest AUC value, of 0.91, with an accuracy value of 95.3%, a sensitivity value of 96.2%, and a specificity value of 94.7% in the testing data set. In contrast, the two physicians yielded an average AUC value of 0.69, an accuracy value of 70.7%, a sensitivity value of 54.4%, and a specificity value of 53.2%. Our findings indicate that the diagnostic performance of deep learning is better than that of physicians in the distinction of PTs from FAs. This further suggests that AI is a valuable tool for aiding clinical diagnosis, thereby advancing precision therapy.

## 1. Introduction

Breast fibroadenomas (FAs) and phyllodes tumors (PTs) are tumors with the same origin: both have fibro and epithelial components in the breast tissue. FAs are the most common benign tumors of breast and completely lack invasiveness. In contrast, despite the low morbidity (0.3% to 1.0%), PTs are potentially invasive in nature [1,2]. The World Health Organization (WHO) has classified PTs as benign, borderline, or malignant on the basis of histological assessments of stromal features, such as hypercellularity, atypia, mitotic activity, overgrowth, and the nature of the tumor borders [3]. Although FAs and PTs have the same histological origin [3], their clinical therapeutic strategies are substantially different. FAs are usually treated nonsurgically or with simple enucleation, while PTs must be resected with an extended margin of at least 1 cm surrounding the mass to avoid local recurrence. This is because PTs have the biological behavior of locally destructive growth and even metastasis. Specifically, previous studies have indicated that the local recurrence is approximately 8%, 21%, and 30% for benign, borderline, and malignant PTs, respectively [4,5,6]. In addition, the incidence of distant metastasis to other organs following malignant PTs is approximately 22% [7]. Therefore, the preoperative diagnosis for differentiating PTs and FAs is critical for tailored treatment.

The three imaging modalities, i.e., ultrasound (US), magnetic resonance imaging (MRI), and mammography, are considered the main preoperational diagnostic methods for breast diseases. USs are more commonly used in China thanks to the high density of breast tissues among Asian women [8]. The US characteristics have been valuable in the differentiation between PTs and FAs. Previous studies suggested that the internal cystic areas and heterogeneous inner echo were typical US features for PTs [9,10]. However, in daily clinical practice, a small and benign borderline or malignant PT can be easily mistaken for an FA, whereas large-volume FAs may show similar visual US features to those of PTs. Although combining certain clinical information and typical sonographic features may raise the confidence of diagnosis, it is still challenging to make a reliable differentiation between PTs and FAs with the naked eye. Moreover, these radiological evaluations are always subjective and lack quantitative metrics. Beyond the imaging diagnosis, cytodiagnosis also encounters difficulty in distinguishing PTs from FAs, because of overlapping pathology features, especially for low-grade PTs [11,12].

With the development of medical image analysis techniques, quantitative methods such as radiomics analysis and AI have been widely used for assisting in the diagnosis of breast diseases. For example, AI has been applied in the differentiation of benign from malignant lesions of breast on MRI [13], predicting the risk of breast cancers from screening mammograms [14], preoperatively predicting the extent of axillary lymph node involvement in early-stage breast cancers [15], predicting neoadjuvant chemotherapy response in breast cancers [16], assessing breast cancer molecular subtypes on the basis of US images [17], and predicting the pathological grade of PTs [18,19]. The application of AI techniques has also been explored in the differential diagnosis of PTs and FAs. Several radiomic studies have shown that the quantitative MRI texture features, combined with clinical characteristics, show better diagnostic performance, which distinguishes PTs from FAs [20,21]. Based on ultrasound images, deep learning has also shown the ability to differentiate between PTs and FAs with good diagnostic accuracy and high negative predictive value (PPV) [22]. Although in these studies, AI shows promise in the distinction of PTs from FAs, the relatively small sample size (fewer than 100 cases) weakens the confidence of the deep-learning method.

In this study, we recruited a cohort of 656 patients with 656 breast lesions and a total of 1945 ultrasound images, and we adopted deep-learning models to differentiate between PTs and FAs. In addition, the ultrasound images were also independently evaluated by two experienced US physicians. We expected that deep learning would show superior performance in distinguishing PTs from FAs compared with US physicians.

## 2. Materials and Methods

### Participants

This retrospective study was approved by the institutional review board of Fudan University Shanghai Cancer Center, which waived the need for informed consent. From January 2015 to February 2022, 656 female patients with the same number of breast lesions (372 FAs and 284 PTs) were retrospectively reviewed for US images and pathological results. Among the 284 PTs, 134 cases were benign PTs; 120 cases were borderline PTs; and 30 cases were malignant PTs. The demographics and clinical characteristics are summarized in Table 1. The inclusion criteria were as follows: (1) all cases were surgically resected and pathologically confirmed; (2) the pathological subtypes of PT (i.e., benign, borderline, and malignant) were definitive; and (3) only grayscale ultrasound images were included. The exclusion criteria were as follows: (1) patients with a maximum tumor diameter less than 5 mm or more than 50 mm [23]; (2) no clear definitions of benign, malignant, and borderline lesions in the pathological diagnosis of PTs; and (3) poor image quality.

**Figure 1:**
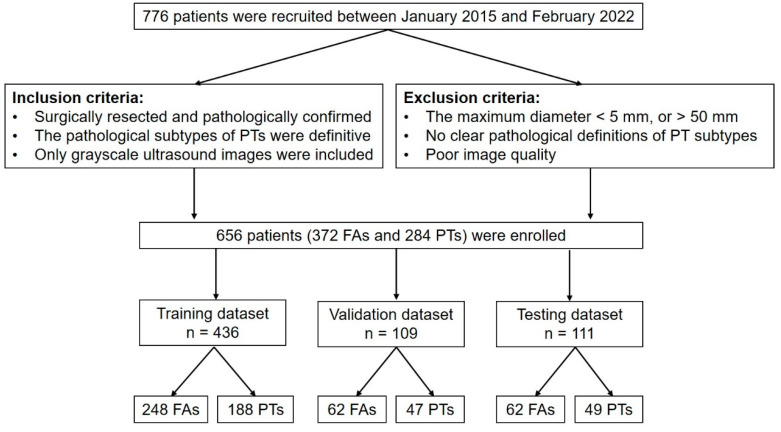
Flowchart of this study. PT = phyllodes tumor; FA = fibroadenoma; n = the number of patients.

We divided the 656 patients into 6 subsets, in which 5 subsets (training data set and validation data set) had 109 patients each and 1 subset (independent testing data set) had 111 patients. Five experiments were conducted. The training data set was used to train the deep-learning models. The independent testing data set was used to verify the performance of all deep-learning models that had been trained. A flowchart of this study is shown in Figure 1.

## 3. US Images Acquisition

The US images were acquired by using the different instruments in our department, including the Philips EPIQ7 and iU22 (Philips Medical Systems, Sydney, Australia); the GE LOGIQ E9, V730, and LOGIQ S8 (GE Healthcare, Chicago, IL, USA), Mindray-Resona7 (Mindray Medical, Shenzhen, China); and the Toshiba-Aplio 500 (Canon Medical Systems, Ohtawara, Japan). All scans were performed with a linear array transducer with a broadband frequency of 5–12 MHz. For larger masses that cannot be completely displayed within the frame, the trapezoidal imaging mode that enlarges the field of view was adopted. For lesions presented in multiple images, all available data were considered, thus resulting in 1217 images of FAs and 728 images of PTs.

### 3.1. Images Preprocessing

**Figure 2:**
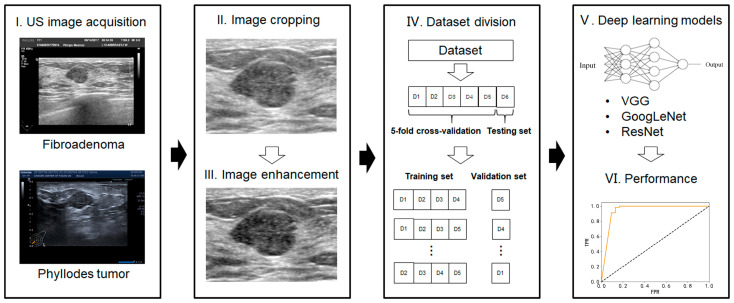
Workflow of the deep-learning model analysis.

All raw DICOM (digital imaging and communication in medicine) images were first converted into JPG format for further processing:

1. Image cropping—in order to avoid the influence of nontumor areas in US images and reduce the computational load, the rectangular ROIs (regions of interest) were manually cropped from raw US images.
2. Image enhancement—the grayscale transformation function was used to enhance the contrast between the foreground and the background of US images. The image enhancement can improve image quality, enrich information, and enhance image interpretation and the recognition effect (Figure 2).

### 3.2. Deep-Learning Models for US Image Analysis

**Figure 3:**
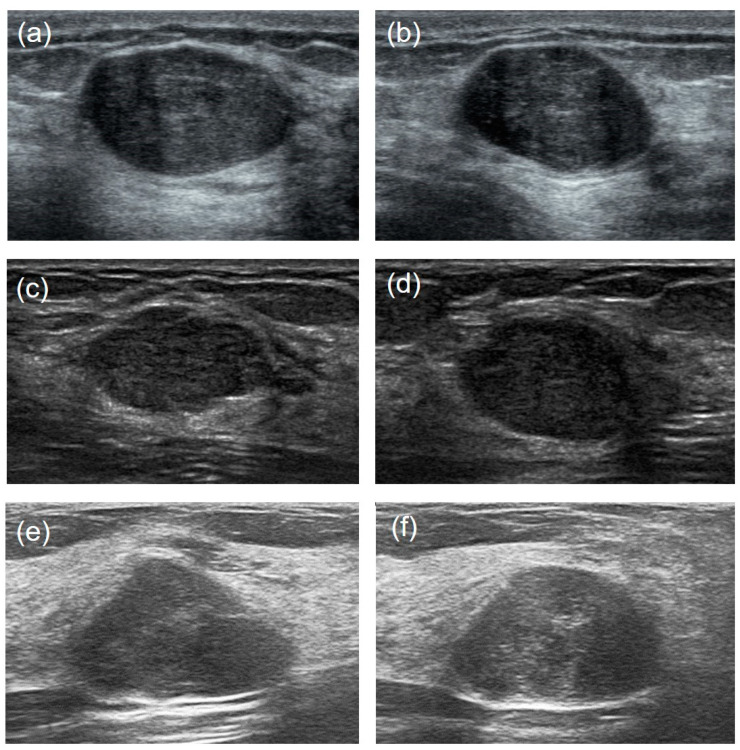
US image comparisons of the FAs and PTs for relatively large lesions. Three couples of similar lesions show an oval hypoechoic mass with circumscribed margins and heterogeneous internal echogenicity. Histopathology confirmed: ((**a**): transverse section, (**b**): longitudinal section) a 44-year-old woman with a 2.1 cm × 1.3 cm × 2.0 cm FA; ((**c**): transverse section, (**d**): longitudinal section) a 35-year-old woman with a benign 1.7 cm × 1.0 cm × 1.5 cm PT; ((**e**): transverse section, (**f**): longitudinal section) a 29-year-old woman with a borderline 2.4 cm × 1.4 cm × 1.7 cm PT.

**Figure 4:**
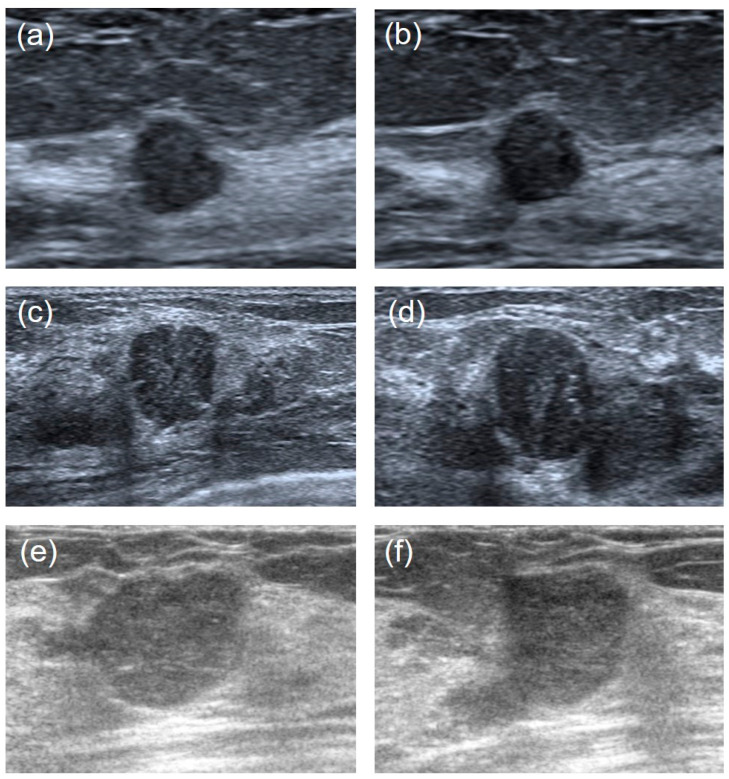
Ultrasound image comparisons of the FAs and PTs for smaller lesions. Three couples of similar lesions showed a round-shaped hypoechoic mass with microlobulated or angular margins and heterogeneous internal echogenicity. Histopathology confirmed: ((**a**): transverse section, (**b**): longitudinal section) a 41-year-old woman with a 0.7 cm × 0.7 cm × 0.5 cm FA. ((**c**): transverse section, (**d**): longitudinal section) a 34-year-old woman with a benign 1.2 cm × 1.3 cm × 1.0 cm PT. ((**e**): transverse section, (**f**): longitudinal section) a 42-years-old woman with a borderline 1.3 cm × 1.4 cm × 1.8 cm PT.

**Figure 5:**
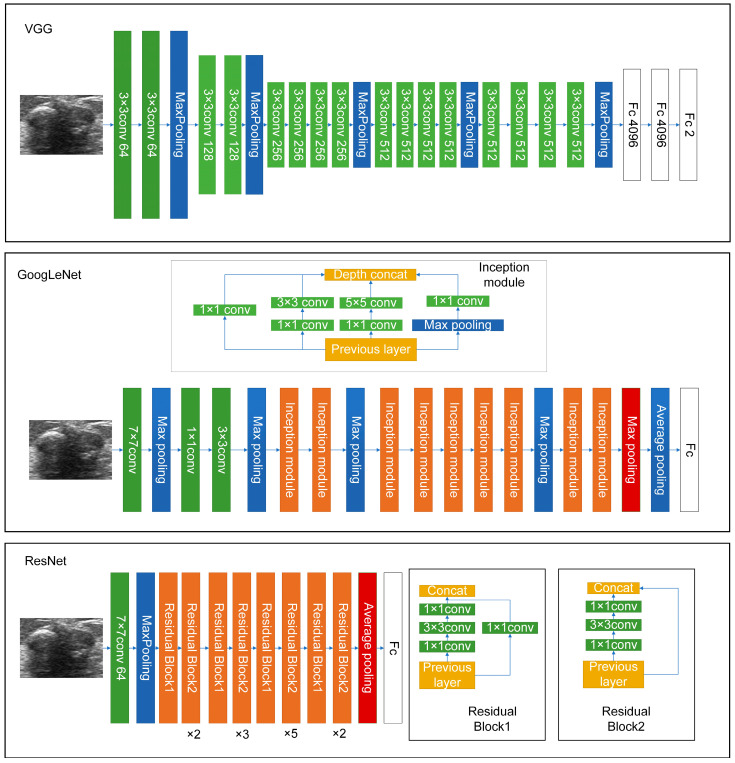
The architecture of the three deep-learning models used in this work.

It is hard to differentiate PTs from FAs because of the high visual similarity in grayscale images of them (see Figure 3 and Figure 4 for representative US images). In order to achieve an optimal image classification, three deep-learning models, including ResNet [24], VGG [25], and GoogLeNet [26], were applied. ResNet was constructed by using residual building blocks, which can effectively solve the problems of gradient disappearance and explosion in convolutional neural networks (CNNs). VGG used a 3 × 3 convolution kernel to stack the neural network and thus deepen the whole neural network. Using a smaller convolution kernel and increasing the depth of the convolutional neural network can improve the performance of the model. GoogLeNet introduced an inception structure (fusion of feature information with different scales) and used a 1 × 1 convolution kernel for dimensionality reduction and mapping. At the same time, the full connection layer was discarded, and the average pooling layer was used, which greatly reduced the model parameters compared with those of VGG. The architecture of the three deep-learning models is shown in Figure 5.

The specific procedures and parameters for deep-learning analysis are as follows: (1) the size of all the images was uniformly adjusted to 224 × 224 pixels; (2) the cross-entropy loss function was selected for the model to calculate the difference between the predicted and real values. The BacthSize was set at 16 in consideration of the computer memory, GPU (graphics processing unit), video memory, sample size of the data set, and input image size; and (3) model validation was conducted with fivefold cross validation. We randomly divided all the patients into five folds for each group (four folds for training and one fold for validation). Figure 2 shows the workflow of our proposed deep-learning model. The model was trained for 30 rounds; that is, all the images were iterated 30 times before the training was completed.

Lesion size, a key feature, was usually measured for radiological diagnoses. In addition, demographic information such as age may also contribute to clinical diagnoses. We therefore reperformed the above deep-learning analysis after including lesion size and age. All the deep-learning models were run on the PyTorch deep-learning framework with a 16-core 3.20 GHz CPU, 16 GB of memory, and a GTX3060 GPU on a Windows 11 system.

### 3.3. Visual Assessment by US Physicians

The images were independently evaluated by two experienced US physicians (physician #1 and physician #2 have 9 and 13 years of experience in assessing breast ultrasounds, respectively). The two physicians were mutually blinded, and both were blinded to previous radiological reports as well as the clinical information of the patients. The breast lesions were visually assessed by physicians on the basis of the Atlas of the Fifth Edition of the Breast Imaging Reporting and Data System (BI-RADS), published by the American College of Radiology [27]. In the present study, the final assessments from the two physicians were made for each breast mass by using dichotomized forms: PT or FA. Given the potential effect of lesion size on the performance of US physicians, all the patients were divided into two subgroups according to lesion size: >2 cm and ≤2 cm. Assessments for these two subgroups were also evaluated.

### 3.4. Statistical Analysis

The statistical analysis was performed using SPSS software (Version 23.0, IBM Corporation). Continuous variables were expressed as mean ± standard deviation and categorical variables as counts (percentage, %). The statistical significance of the categorical variables was evaluated by using chi-square tests, and the variables were continuously evaluated by using independent sample *t*-tests. In all the analyses, a *p*-value less than 0.05 was considered statistically significant. The diagnostic performance values of the deep-learning models were assessed by using receiver operating characteristic (ROC) analysis and the area under the curve (AUC), accuracy, sensitivity, specificity, PPV, and NPV. For the US physicians’ diagnoses, the AUC, accuracy, sensitivity, specificity, PPV, and NPV were likewise calculated.

## 4. Results

A significant difference in age was found between patients with PTs (46.4 ± 10.9 years) and FAs (40.1 ± 12.9 years) (*p* < 0.0001). A significant difference in tumor size was also detected between PTs (29.1 ± 9.9 mm) and FAs (18.9 ± 7.9 mm) (*p* < 0.0001). The BI-RADS categories of PTs were higher than those of FAs (*p* < 0.0001).

The diagnostic performance of the three deep-learning models in the fivefold cross validation is shown in Table 2. Specifically, the performance of ResNet in the training data set showed the highest accuracy, at 94.3%, 95.2%, 94.7%, 95.0%, and 94.3%, respectively; and the accuracy was 96.3%, 92.7%, 94.5%, 93.6%, and 96.3% in the validation data set, respectively. The accuracy of GoogLeNet and VGG ranged from 66.1% to 84.6% in the training data set, and ranged from 62.4% to 81.5% in the validation data set.

**Figure 6:**
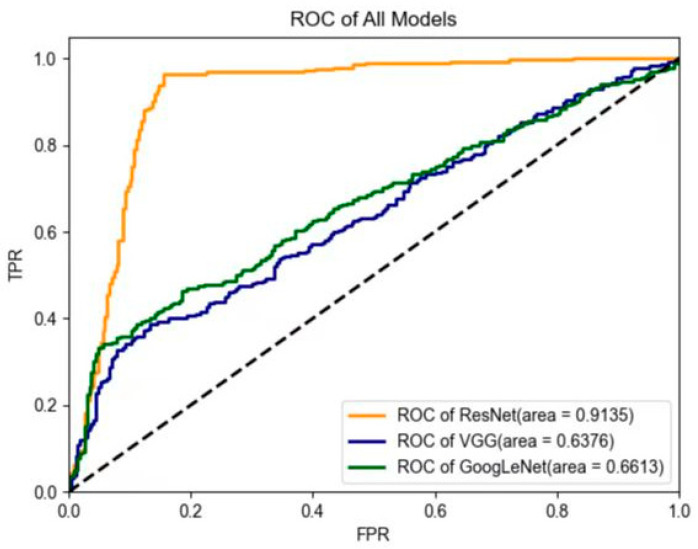
Comparison of receiver operating characteristic (ROC) curves between the three models in the testing data set. In contrast, the two US physicians yielded an average AUC value of 0.69 (for physician #1: AUC = 0.70; for physician #2: AUC = 0.68). The average diagnostic accuracy, sensitivity, specificity, PPV, and NPV for the two US physicians were 70.7%, 54.4%, 83.2%, 73.3%, and 70.7%, respectively (for physician #1: accuracy = 72.6%; sensitivity = 47.2%; specificity = 91.9%; PPV = 81.7%; and NPV = 69.5%; for physician #2: accuracy = 68.9%; sensitivity = 61.6%; specificity = 74.5%; PPV = 64.8%; and NPV = 71.8%) (Table 4). After the patients were further divided into two subgroups according to lesion size, the US physicians showed low sensitivity (25.4%) and high specificity (88.3%) for lesions less than 2 cm; conversely, the sensitivity (64.1%) increased and the specificity (73.2%) decreased when the tumor size was larger than 2 cm.

The performance of three deep-learning models in the testing data set is shown in Table 3. ResNet had the best diagnostic performance, with an AUC value of 0.91, an accuracy value of 95.3%, a sensitivity value of 96.2%, a specificity value of 94.7%, a PPV of 93.1%, and an NPV of 97.1% in the testing data set. GoogLeNet yielded an AUC value of 0.66, an accuracy value of 76.0%, a sensitivity value of 66.6%, a specificity value of 96.1%, a PPV of 97.1%, and an NPV of 59.4%. Lastly, VGG yielded an AUC value of 0.64, an accuracy value of 73.7%, a sensitivity value of 63.6%, a specificity value of 94.4%, a PPV of 95.1%, and an NPV of 56.8% in the testing data set. The ROC curves of the deep-learning models are shown in Figure 6. In addition, after including lesion size and age in the ResNet model, we found a slightly enhanced diagnostic performance value, specifically an AUC value of 0.94, an accuracy value of 96.4%, a sensitivity value of 95.9%, and a specificity value of 96.8% in the testing data set.

## 5. Discussion

The differential diagnosis of PTs and FAs is clinically important for breast surgeons to determine appropriate surgical plans. Meanwhile, it is also beneficial for patients to avoid a second surgery when a PT is misdiagnosed as an FA. As PTs and FAs have the same cellular origin, their imaging appearance is quite similar, and therefore, the exact differentiation is challenging for radiologists. In recent years, AI has shown that it can play a promising role in medical image analysis, particularly for aiding clinical diagnosis. Thanks to its great performance, increasing studies have used deep learning to help in the diagnostic classification of PTs and FAs. However, in these previous studies, only a limited number of patients were included, in particular those who had PTs, because of the low prevalence of PTs [20,22,23]. For example, a previous deep-learning study of 26 patients showed that AUC for a differential diagnosis of FA and PT was 0.73 [22]. In the present study, with a relatively large data set of US images and a comparable number of patients for the two categories (i.e., 284 patients with PTs and 372 patients with FAs), three deep-learning models were applied for the differential diagnosis of PTs and FAs. We found that the AUC values were 0.91, 0.66, and 0.64 for ResNet, GoogLeNet, and VGG, respectively. Although the diagnostic performance was not the poorest with the small data set, sample size may affect the reliability of classification, especially for deep-learning models.

Among the three models, we found that the ResNet model showed the best performance in diagnosing FA and PT in ultrasound images. This is probably attributed to the differences in the model structure and the internal modules [24]. First, the ResNet model used a deeper and wider network structure than GoogLeNet and VGG did, which can effectively improve network performance. Second, ResNet used the residual structure to mitigate degradation problems, which derived from the increase of the number of layers. Finally, ResNet used batch normalization to solve the problem of gradient disappearance or gradient explosion, which can effectively prevent the decline of accuracy. Given that no other classification tasks were tested in current study, our finding may be not generalized to other classification problems.

The performance of the ResNet model surpassed that of the two experienced US physicians. Previous studies have reported that it is very difficult for US physicians to distinguish the subtle differences in the grayscale images because of the overlapping features of FAs and PTs [28,29]. AI can extract objective and quantitative image information from lesions thanks to its powerful computing and learning abilities, which makes it superior to human experience [30]. However, a deep-learning study of 26 patients showed that the PPV of radiologists was better than that of deep learning for the diagnosis of PTs and FAs [22]. In contrast, our results with three deep-learning models consistently revealed that the PPV of the deep-learning model was superior to that of US physicians. This further suggests that the number of patients may bias the diagnostic performance. Importantly, the two US physicians were experts working at the top-ranked cancer hospital in China with a huge number of outpatients who helped them accumulate rich experiences in breast diseases. This not only further suggests the outperformance of the deep-learning model but also implies its potential value in helping less-experienced US physicians.

Beyond the information from ultrasound images, demographic characteristics also contributed to the differentiation between PTs and FAs. Our results showed that the performance of the ResNet deep-learning model was slightly improved after incorporating age and tumor size into the model. Prior studies showed that age was statistically associated with the occurrence of PTs [20,31]. We noticed that the average age of a PT patient (46.4 ± 10.9 years) was significantly higher than that of an FA patient (40.1 ± 12.9 years), which aligns with the details of previous studies. This may explain the improved performance after including age in the ResNet model. Tumor size is one of the key factors that must be considered in the differential diagnosis of PTs and FAs [10,20,32,33]. In accordance with previous studies, we found that PTs are usually larger than FAs in tumor size. The performance of US physicians was tumor size dependent. When patients were divided into two subgroups by using a cutoff of 2 cm, the US physicians showed low sensitivity and high specificity for lesions less than 2 cm; conversely, the sensitivity increased and the specificity decreased when the tumor size was larger than 2 cm. This indicates that tumor size should be considered when using AI algorithms to differentiate PTs from FAs.

Our study has several limitations. First, any retrospective single-center study may have a selection bias and may need to be validated with other comprehensive external cohorts to determine the value of the model in clinical practice and improve confidence in its performance. Second, the model was trained to distinguish between two types of breast masses, PTs and FAs. However, it may be not generalized for the differential diagnosis of other breast diseases, such as invasive cancers or inflammation. Moreover, the differential diagnosis for different pathological types of PTs was not studied. This will be considered in the future. Third, deep-learning approaches have been widely used in medical image analysis. Although many models might be effective for classification problems, the purpose of this study is to explore whether deep-learning approaches can make better differential diagnoses of FAs and PTs compared with those of ultrasound physicians. Accordingly, we adopted three commonly used deep-learning models with the same loss function for the differential diagnosis of FAs and PTs and found that the ResNet model showed a great classification performance value (ACC of ~95%). Therefore, no other models, such as deep distance learning [34], were included in this study. Lastly, the AUC values for the deep-learning models and the US physicians were difficult to directly compare because they were acquired with different algorithms.

## 6. Conclusions

In conclusion, our study demonstrates that the deep-learning ResNet model based on US images is superior to experienced US physicians in differentiating PTs from FAs and may serve as a complementary tool to assist in clinical decision-making by US physicians.

## Figures and Tables

**Table 1:** Demographics and clinical characteristics of patients.

Characteristics	PT (*n* = 284)	FA (*n* = 372)	T/χ^2^ Value	*p*-Value
Age (years)	46.4 ± 10.9	40.1 ± 12.9	6.6	<0.0001
Max diameter (mm)	29.1 ± 9.9	18.9 ± 7.9	15.2	<0.0001
BI-RADS categories:			61.1	<0.0001
3	56 (20%)	76 (20%)		
4A	158 (56%)	280 (75%)		
4B	57 (20%)	15 (4%)		
4C	10 (3.5%)	1 (0.3%)		
5	3 (1%)	0 (0%)		
Pathological types of PTs:				
Benign	134 (47.2%)			
Borderline	120 (42.3%)			
Malignant	30 (10.5%)			

Note: PT = phyllodes tumor; FA = fibroadenoma; BI-RADS = Breast Imaging Reporting and Data System, *n* = the number of patients.

**Table 2:** The performance of three deep-learning models in the fivefold cross validation.

Data Sets	Models	ACC1 (%)	ACC2 (%)	ACC3 (%)	ACC4 (%)	ACC5 (%)
Training (*n* = 436)	ResNet	94.3 (90.0–96.8)	95.2 (92.1–97.3)	94.7 (91.6–97.7)	95.0 (91.9–98.1)	94.3 (91.0–97.5)
	GoogLeNet	83.9 (79.6–87.8)	81.9 (77.5–86.1)	83.5 (79.2–87.1)	84.6 (80.3–88.0)	84.4 (80.1–87.9)
	VGG	66.1 (60.4–71.8)	67.0 (61.3–72.7)	66.3 (60.6–72.0)	67.4 (61.7–73.1)	65.4 (59.6–71.2)
Validation (*n* = 109)	ResNet	96.3 (90.5–99.2)	92.7 (85.3–97.0)	94.5 (88.8–98.3)	93.6 (86.8–98.1)	96.3 (88.8–98.1)
	GoogLeNet	78.9 (68.8–85.7)	81.5 (73.3–89.7)	78.0 (66.7–89.3)	78.9 (67.8–90.0)	78.9 (67.8–90.0)
	VGG	65.1 (53.4–76.8)	62.4 (50.4–74.4)	65.1 (53.4–76.8)	67.0 (55.2–78.8)	66.1 (54.4–77.8)

Note: ACC = accuracy, *n* = the number of patients, and the 95% confidence intervals are in parenthesis.

**Table 3:** The performance comparison of different models for the testing data set.

Models	AUC	ACC (%)	SENS (%)	SPEC (%)	PPV (%)	NPV (%)
ResNet	0.91 (0.88–0.95)	95.3 (91.4–99.3)	96.2 (92.5–99.8)	94.7 (89.6–99.9)	93.1 (77.1–98.6)	97.1 (77.2–99.6)
GoogLeNet	0.66 (0.59–0.73)	76.0 (61.4–90.6)	66.6 (48.3–84.9)	96.1 (93.0–99.0)	97.1 (94.8–99.5)	59.4 (30.6–88.1)
VGG	0.64 (0.58–0.69)	73.7 (69.2–78.2)	63.6 (58.5–68.8)	94.4 (66.5–99.3)	95.1 (90.8–99.9)	56.8 (43.2–70.3)

Note: AUC = area under the curve, ACC = accuracy, SENS = sensitivity, SPEC = specificity, PPV = positive predictive value, NPV = negative predictive value, and the 95% confidence intervals are in parenthesis.

**Table 4:** Visual assessment of US images by physicians.

Physicians	AUC	ACC (%)	SENS (%)	SPEC (%)	PPV (%)	NPV (%)
Physician #1	0.70 (0.65–0.74)	72.6 (65.6–79.4)	47.2 (40.1–54.8)	91.9 (85.9–97.4)	81.7 (72.8–89.3)	69.5 (61.4–76.3)
Physician #2	0.68 (0.64–0.72)	68.9 (62.7–75.1)	61.6 (54.1–68.7)	74.5 (67.7–80.7)	64.8 (56.7–71.2)	71.8 (65.8–77.3)
Average	0.69	70.7	54.4	83.2	73.3	70.7

Note: AUC = area under the curve, ACC = accuracy, SENS = sensitivity, SPEC = specificity, PPV = positive predictive value, NPV = negative predictive value, and the 95% confidence intervals are in parenthesis.

## Data Availability

All the imaging data can be provided upon reasonable request to the corresponding author.

## Abbreviations

The following abbreviations are used in this manuscript:

ACC	Accuracy
AI	Artificial intelligence
AUC	Area under the curve
BI-RADS	Breast Imaging Reporting and Data System
DICOM	Digital imaging and communication in medicine
FA	Fibroadenoma
MRI	Magnetic resonance imaging
NPV	Negative predictive value
PPV	Positive predictive value
PT	Phyllodes tumor
ROC	Receiver operating characteristic
ROI	Regions of interest
SENS	Sensitivity
SPEC	Specificity
US	Ultrasound
